# Hepatocellular Carcinoma with Vascular and Cardiac Involvement in a Young Patient with Non-Cirrhotic Hepatitis B: A Case Report

**DOI:** 10.1159/000539093

**Published:** 2024-07-26

**Authors:** Inês Botto, Juliana Serrazina, Carlos Rodrigues Freitas, Sofia Carvalhana, Gonçalo Nogueira-Costa, Helena Cortez-Pinto

**Affiliations:** a Serviço de Gastrenterologia e Hepatologia, Unidade Local de Saúde de Santa Maria, Lisbon, Portugal; b Serviço de Oncologia, Unidade Local de Saúde de Santa Maria, Lisbon, Portugal; c Faculdade de Medicina, Universidade de Lisboa, Lisbon, Portugal

**Keywords:** Hepatocellular carcinoma, Hepatitis B virus, Right atrial extension, Carcinoma hepatocelular, Vírus da hepatite B, Metastização auricular

## Abstract

**Introduction:**

Hepatocellular carcinoma (HCC) is a highly vascular malignancy with the potential for intravascular invasion. However, vascular extension into the cardiac chambers is extremely rare.

**Case Presentation:**

We present a 25-year-old male patient in whom the investigation of a cardiac murmur led to the discovery of an intracardiac mass that proved to be advanced-stage HCC with vascular invasion of the right heart. The patient had a previously unknown non-cirrhotic chronic hepatitis B virus (HBV) infection. Despite antiviral therapy and systemic treatment for HCC, he eventually died about 1 month later due to disease progression.

**Conclusion:**

This report highlights the importance of early HBV diagnosis and treatment for timely detection and management of HCC. Advanced-stage HCC, particularly with cardiac involvement, has an extremely poor prognosis.

## Resumo

**Introdução:**

O carcinoma hepatocelular é uma neoplasia com elevado risco de invasão vascular, porém, a invasão cardíaca está raramente descrita.

**Descrição do caso:**

Descreve-se um caso de um doente, do sexo masculino, de 25 anos, sem antecedentes de cirrose hepática, que se apresentou com queixas de cansaço, dispneia e perda ponderal. A investigação adicional mostrou um sopro cardíaco, resultante da invasão cardíaca direita por carcinoma hepatocelular e hepatite B crónica. Apesar da instituição da terapêutica dirigida ao carcinoma hepatocelular e terapêutica antiviral, verificou-se progressão da doença levando ao falecimento do doente um mês após o diagnóstico.

**Conclusão:**

Este caso destaca a importância do diagnóstico e tratamento precoces da hepatite B crónica, potenciando a deteção atempada do carcinoma hepatocelular. O carcinoma hepatocelular em estadio avançado, nomeadamente com invasão cardíaca, está associado a mau prognóstico.

## Introduction

Hepatocellular carcinoma (HCC) is the sixth-most common cancer worldwide and accounts for 90% of all primary liver cancers [1]. Over 90% of HCC cases occur in the setting of chronic liver disease, with cirrhosis from several etiologies as the primary risk factor. Hepatitis B virus (HBV) infection is documented in approximately 50% of HCC cases [2]. HCC metastasis can occur in different locations, with the most affected sites being the lungs, lymph nodes, and bones. Despite being a highly vascular tumor with intravascular dissemination, cardiac involvement is a rare manifestation (1–4%) and is usually diagnosed in patients with known HCC during oncology follow-up or as an autopsy finding [3].

## Case Report

**Fig. 1.**
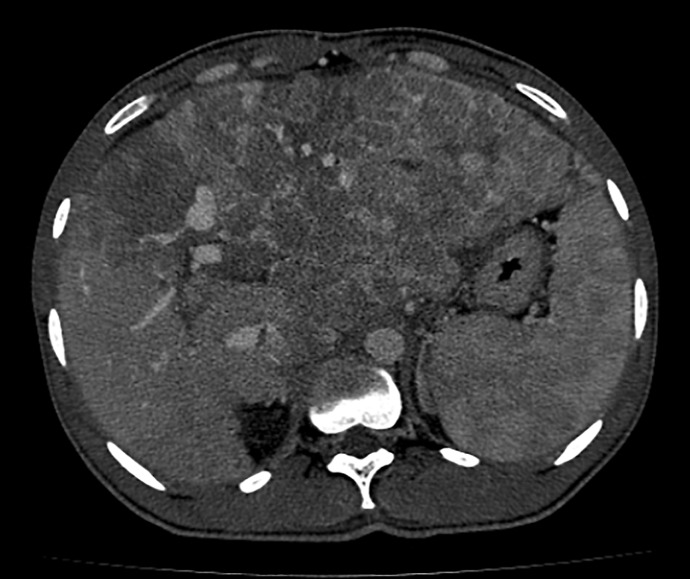
CT scan of the abdomen. Heterogeneous hepatomegaly with multiple coalescent lesions involving most of the left and caudate lobes and also the right lobe, with arterial enhancement and washout, suggestive of multicentric HCC. CT, computed tomography.

**Fig. 2.**
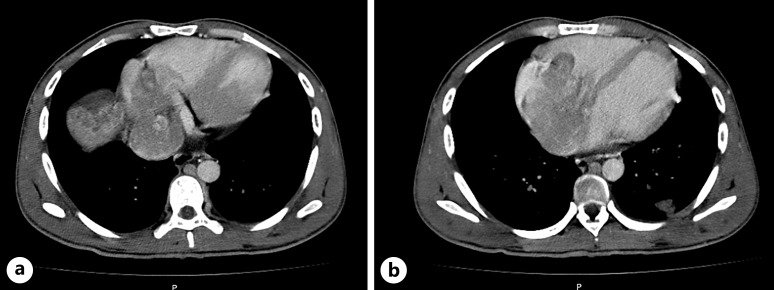
CT scan of the thorax. **a** Macrovascular invasion of the left portal vein, middle suprahepatic veins, and inferior vena cava. **b** Tumoral extension into the lumen of the right ventricle and atrium, with an intracardiac component measuring 9 × 7.5 × 6 mm (AP × T × L). CT, computed tomography.

A 25-year-old male patient from Guinea, in Portugal for 6 months, with no known medical history presented with a 2-week history of fatigue, exertional dyspnea, anorexia, and weight loss. He also mentioned abdominal pain for the last 4 days. The patient consumed 20 g of alcohol per week and had no history of drug consumption or tobacco smoking habits. He was employed as a construction worker and did not have any relevant travel history. Physical examination showed a systolic murmur in the tricuspid area, an enlarged liver and right upper quadrant pain, with no ascites or signs of chronic liver disease stigmata. The electrocardiogram revealed sinus tachycardia with a right bundle branch block and the chest radiograph showed an enlarged cardiac silhouette. An echocardiogram showed an intracardial mass in the right atrium (RA) (80 mm × 64 mm), obstructing the tricuspid valve and causing functional stenosis. His laboratory tests showed normocytic anemia (hemoglobin 11.6 g/dL [ref. 13–17.5]), slightly elevated liver enzymes (AST 91 U/L [ref. 0–34], ALT 68 U/L [ref. 10–49], GGT 68 U/L [ref. 0–60], ALP 393 U/L [ref. 35–105]), with normal bilirubin (1.12 mg/dL [ref. <1.2]), and elevated NT-proBNP (457 pg/mL [ref. <300]). He underwent a computed tomography scan of the thorax, abdomen, and pelvis, which revealed multiple coalescent lesions evolving mainly the left and caudate lobes, consistent with multicentric HCC (shown in Fig. 1). Additionally, macrovascular invasion was observed in the left portal vein, middle suprahepatic veins, and inferior vena cava, extending into the lumen of the right ventricle and atrium. The intracardiac component measured 9 × 7.5 × 6 mm (AP × T × L) (shown in Fig. 2). The computed tomography scan also identified bilateral pulmonary secondary lesions and signs of multiple segmental and sub-segmental bilateral pulmonary embolisms. Further investigation revealed an HBV infection (AgHbs positive, anti-Hbs negative, AgHbe positive, anti-Hbe negative) with a viral load of 1,160,000 IU/mL and an alpha-fetoprotein of 357,440 ng/mL (ref. <7). The patient was classified as Barcelona Clinic Liver Cancer (BCLC) Stage C due to the presence of extra-hepatic spread and portal invasion.

After being discussed at a multidisciplinary team meeting, the patient was initiated on a regimen of tenofovir (300 mg/day) and sorafenib (400 mg/day). A few days later, the patient developed gastrointestinal side effects and stopped therapy. Due to rapid clinical and liver function deterioration, a palliative option was considered, and he died 1 month after the diagnosis of disease progression.

## Conclusion

It is estimated that 854,000 new cases of HCC are diagnosed per year, with a growing incidence worldwide, making HCC a major global health problem [1]. In approximately 50% of HCC cases worldwide there is a subjacent HBV infection [2]. Although cirrhosis is considered the primary risk factor for HCC, chronic hepatitis B has carcinogenic potential itself by several mutagenetic routes, and up to 30% of chronic hepatitis B-related HCCs arise in non-cirrhotic liver, mainly in Asian patients, and patients with a family history of HCC. Therefore, HCC surveillance is recommended in European guidelines not only in cirrhotic patients but also in hepatitis B carriers with risk factors (family history of HCC or Asian patients over 40 or 50 years old, in men and women, respectively) [1]. On the other hand, in 2019, the burden of HBV infection was significant globally, with an estimated all-age prevalence of chronic HBV infection at 4.1%, corresponding to approximately 316 million infected individuals [4]. Recent recommendations from the Centers for Disease Control and Prevention (CDC) suggest HBV screening at least once during a lifetime for adults aged ≥18 years [5].

The prevalence of metastatic disease in HCC has been reported to be 11.2–25.5%, with the lungs (55%), lymph nodes (41%) and bones (7%) being the most commonly affected sites [6]. HCC has a strong tendency for macrovascular invasion, with a high incidence of portal and hepatic vein thrombosis (35–44% and 2–12%, respectively) [7].

Direct tumor extension from the hepatic veins into the inferior vena cava and the RA is the primary mechanism of cardiac involvement in HCC, with a reported incidence of 1.4–4.9% [8]. Jun et al. [9] analyzed a cohort of 665 patients with HCC, 33 of which had RA invasion, and identified the following risk factors for its extension: invasion of the hepatic vein, simultaneous invasion of the portal vein and the inferior vena cava, the presence of multinodular HCC and advanced disease stage (classified as modified TNM staging ≥ Iva). Tumor size and elevated alpha-fetoprotein levels are also associated with vascular invasion [10]. The clinical presentation of HCC with RA extension is primarily influenced by the size of the intracardiac component and can range from asymptomatic (39.5%) to bilateral lower limb edema (37.5%) and exertional dyspnea [11].

In this case, considering the presence of pulmonary metastasis and extensive liver involvement, systemic treatment emerged as the optimal option. However, due to the presence of pulmonary thromboembolism and the need for anticoagulation, as well as the presence of active hepatitis B infection, the utilization of bevacizumab-atezolizumab was precluded. Following the development of gastrointestinal intolerance to sorafenib, alternative therapies such as ramucirumab were considered. However, the deterioration in clinical functional status and liver function led to the progression to BCLC-D staging, preventing the initiation of a second-line systemic therapy. While aggressive approaches and multimodality have been reported in clinical cases, when HCC presents with cardiac involvement, it significantly restricts therapeutic options, and there is no consensus regarding the optimal treatment. In the absence of extra-hepatic spread, transarterial chemoembolization (TACE) has been documented in earlier stages of HCC with atrial macrovascular invasion [12, 13]. Kolarich et al. [13] reported a retrospective series of 8 patients with macrovascular invasion treated with TACE, supplemented by lenvatinib in 4 cases, resulting in an 86 ± 19% reduction in intra-atrial tumor burden. Although surgery has shown good technical success, even in cases with extra-hepatic metastasis in patients with good hepatic reserve, with a 1-year survival of 29.2% in those undergoing non-curative surgery [14], the multicentric nature of the tumor burden precluded surgical intervention in our case. Wakayama et al. [14] described a retrospective cohort of 5 patients who underwent curative resection, all of whom experienced postoperative recurrence, with a median recurrence-free survival of 3.8 months. More recently, a case report detailed a HCC with RA involvement managed with multimodal therapy, incorporating systemic therapy followed by TACE and liver resection [15]. Despite the documentation of more aggressive and multimodality treatments in the literature, evidence regarding their efficacy and impact on survival remains sparse.

The prognosis for HCC with intra-cardiac involvement remains poor, with a median survival range of 1–4 months [15]. Cardiopulmonary complications include pulmonary embolism, heart failure, life-threatening arrhythmias, and systemic metastasis, with heart failure and sudden cardiac death being the primary causes of mortality, affecting up to 25% of patients [15].

The severity and complexity of the case, combined with the patient’s young age, make this an ethically challenging situation. The multidisciplinary interaction with oncology and palliative care was extremely important, not only for therapeutic decisions but also to ensure a dignified end of life.

This case report presents a rare case of HCC with extensive vascular and cardiac involvement in a young patient with previously unknown non-cirrhotic HBV infection. The presentation with mainly cardiac symptoms and the investigation of a cardiac mass that led to the diagnosis makes this case unusual and interesting. Furthermore, it highlights the importance of HBV screening and diagnosis since it may lead to timely detection of HCC – a crucial step in averting diagnosis at advanced stages.

We acknowledge the limitations of a case report approach such as the lack of ability to generalize or to infer a cause-effect association. Particularly in this rare presentation of HCC with cardiac involvement, further research is needed to determine the optimal approach to this complex situation.

## Acknowledgment

The authors acknowledge the invaluable contributions and discussions with all members of the medical and nursing team involved in the care of this patient, particularly the multidisciplinary liver cancer team, whose invaluable contributions enhanced the management of this case.

## Statement of Ethics

The authors have no conflicts of ethics. Ethical approval was not required for this study in accordance with local/national guidelines. A written informed consent was obtained from participants for publication of the details of their medical case and any accompanying images.

## Conflict of Interest Statement

The authors have no conflicts of interest to declare.

## Funding Sources

There were no funding sources relevant to this case report.

## Author Contributions

Inês Botto and Juliana Serrazina (shared co-first authorship): drafting and preparation of the case report, literature research, and acquisition and interpretation of clinical data for the case report. Sofia Carvalhana, Carlos Freitas, Gonçalo Nogueira-Costa, and Helena Cortez Pinto: critically revising the case report and approving the final manuscript.

## Data Availability

All data generated or analyzed during this study are included in this article and/or its supplementary material files. Further inquiries can be directed to the corresponding author.

## References

[1] European Association for the Study of the Liver . EASL clinical practice guidelines: management of hepatocellular carcinoma. J Hepatol. 2018;69(1):182–236. 29628281 10.1016/j.jhep.2018.03.019

[2] Llovet JM , Kelley RK, Villanueva A, Singal AG, Pikarsky E, Roayaie S, . Hepatocellular carcinoma. Nat Rev Dis Primers. 2021;7(1):6. 33479224 10.1038/s41572-020-00240-3 PMC13537433

[3] Legris V , Sergerie M, Garceau P, Thibodeau-Jarry N. A right atrial mass as initial presentation of a hepatocellular carcinoma. CJC Open. 2021;3(3):376–8. 33778456 10.1016/j.cjco.2020.11.001 PMC7985016

[4] GBD 2019 Hepatitis B Collaborators . Global, regional, and national burden of hepatitis B, 1990-2019: a systematic analysis for the Global Burden of Disease Study 2019. Lancet Gastroenterol Hepatol. 2022;7(9):796–829. 35738290 10.1016/S2468-1253(22)00124-8 PMC9349325

[5] Conners EE , Panagiotakopoulos L, Hofmeister MG, Spradling PR, Hagan LM, Harris AM, . (2023). Screening and testing for hepatitis B Virus infection: CDC recommendations – United States, 2023. MMWR Recomm Rep. 2023;72(1):1–25. 10.15585/mmwr.rr7201a1 PMC9997714 36893044

[6] Becker AK , Tso DK, Harris AC, Malfair D, Chang SD. Extrahepatic metastases of hepatocellular carcinoma: a spectrum of imaging findings. Can Assoc Radiol J. 2014;65(1):60–6. 24239313 10.1016/j.carj.2013.05.004

[7] Quirk M , Kim YH, Saab S, Lee EW. Management of hepatocellular carcinoma with portal vein thrombosis. World J Gastroenterol. 2015;21(12):3462–71. 25834310 10.3748/wjg.v21.i12.3462 PMC4375567

[8] Dantas E , Matos D, Coelho M, Sequeira C, Cardoso C, Oliveira AP. Hepatocellular carcinoma with atrial extension: a case report. GE Port J Gastroenterol. 2021;28(5):360–3. 34604468 10.1159/000511643 PMC8443931

[9] Jun CH , Sim DW, Kim SH, Hong HJ, Chung MW, Cho SB, . Risk factors for patients with stage IVB hepatocellular carcinoma and extension into the heart: prognostic and therapeutic implications. Yonsei Med J. 2014;55(2):379–86. 24532507 10.3349/ymj.2014.55.2.379 PMC3936619

[10] Sakata J , Shirai Y, Wakai T, Kaneko K, Nagahashi M, Hatakeyama K. Preoperative predictors of vascular invasion in hepatocellular carcinoma. Eur J Surg Oncol. 2008;34(8):900–5. 18343084 10.1016/j.ejso.2008.01.031

[11] Liu YC , Ho YL, Huang GT, Chen DS, Sheu JC, Chen CH. Clinical manifestations and survival of patients with hepatocellular carcinoma and cardiac metastasis. J Gastroenterol Hepatol. 2010;25(1):150–5. 19929928 10.1111/j.1440-1746.2009.06036.x

[12] Kamal MW , Farshidpour M, Long AW, Farooqui S, Cunningham SC. Hepatocellular carcinoma with intra-atrial extension responding to transarterial chemoembolization via the right hepatic and right inferior phrenic arteries. Gastrointest Cancer Res. 2014;7(3–4):111–6. 25276266 PMC4171979

[13] Kolarich A , Frangakis C, Yarchoan M, Hong K, Georgiades C. Transarterial chemoembolization in patients with hepatocellular carcinoma with intra-atrial tumor extension: imaging response and oncologic outcomes. J Vasc Interv Radiol. 2021;32(8):1203–8.e1. 34332718 10.1016/j.jvir.2021.04.012 PMC9472765

[14] Wakayama K , Kamiyama T, Yokoo H, Kakisaka T, Kamachi H, Tsuruga Y, . Surgical management of hepatocellular carcinoma with tumor thrombi in the inferior vena cava or right atrium. World J Surg Oncol. 2013;11:259. 24093164 10.1186/1477-7819-11-259 PMC3851861

[15] Liu J , Zhang RX, Dong B, Guo K, Gao ZM, Wang LM. Hepatocellular carcinoma with inferior vena cava and right atrium thrombus: a case report. World J Clin Cases. 2021;9(26):7893–900. 34621843 10.12998/wjcc.v9.i26.7893 PMC8462251

